# Empowerment: A Framework to Develop Advocacy in African American Grandmothers Providing Care for Their Grandchildren

**DOI:** 10.5402/2011/531717

**Published:** 2011-05-19

**Authors:** Gloria F. Carr

**Affiliations:** Loewenberg School of Nursing, The University of Memphis, 610 Goodman Street, Memphis, TN 38152, USA

## Abstract

*Purpose*. Based on a review of the literature, this paper presents a unique and innovative model that offers an empowerment framework, which may be used to develop advocacy in African American (AA) grandmother caregivers. This proposed framework centers on education as a catalyst to the empowerment process in these grandmothers. Application of this model has potential to guide the practice of healthcare providers as they assist these caregivers in managing their own lives. *Methodology*. Various empowerment definitions and research were used to develop this empowerment framework. *Discussion*. This framework offers an empowerment education program for AA grandmothers providing care for their grandchildren on topics that they feel are necessary to appropriately care for themselves and their grandchildren. Outcomes of this empowerment education are to develop skills within these grandmothers so that they will be able to advocate for themselves, their grandchildren, and others within their communities. This education will ultimately produce skillful AA grandmothers who will develop abilities to empower themselves and other AA grandmothers who are in similar circumstances.

## 1. Introduction

For almost two decades, grandparents have increasingly become primary caregivers for their grandchildren. Grandparent caregivers experience altered health outcomes secondary to the stress of stretched finances and other problems—when caregiving roles are assumed, grandparents are often pushed into poverty [1–5]. In fact, approximately 19% of grandparent caregiver families are below the poverty line [6]. 

The first discipline within healthcare to examine empowerment among its consumers, psychology investigated empowerment in the context of participatory effort for individuals to gain control of themselves and their environment [7]. Empowerment has also been contextualized among healthcare providers desiring to empower patients by sharing information that encourages patient participation in their own well-being [8–12]. Recently, individuals were empowered though education to build advocacy skills [13] and to offer empowerment as a framework to induce advocacy to foster self-efficacy in terms of personal and community benefits [14].

This paper describes an empowerment model for use and implementation among African American (AA) grandmothers providing care for their grandchildren. This model is discussed in consideration of definitions and historical contexts; empowerment research, including causal factors and severity levels; an empowerment theory and theoretical framework; implications, gaps in the research, and future recommendations.

## 2. Definitions and Historical Context

### 2.1. Definitions

Empowerment is a concept that extends across boundaries of many disciplines: psychology, healthcare, nursing, and industry. Definitions, therefore, have been designed to accommodate various populations and offered in social and individual contexts [15, 16]. Zimmerman and Rappaport [16, page 725] describe empowerment as “the ability of individuals to gain control socially, politically, economically, and psychologically through access to information, knowledge and skills, decision-making, individual self-efficacy, community participation, and perceived control” while Gibson [15, page 354] defines it as “a process of helping people to assert control over the factors which affect their lives. This process encompasses both the individual responsibility in healthcare and the broader institutional, organizational, or societal responsibilities in enabling people to assume responsibility for their own health.” 

Empowerment has also been defined as “a process of enhancing feelings of self-efficacy among organizational members through the identification of conditions that foster powerlessness and through their removal by both formal organizational practices and informal techniques providing efficacy information” [17, page 474]. Moreover, empowerment has been defined as an outcome [7, 18, 19]. This outcome is evidenced by enhanced autonomy, communication, decision-making ability, and advocacy skills [18, 20]. 

Empowerment then is a process of nurturing opportunities for people to take ownership in becoming self-sufficient, self-confident, and self-supporting in social, political, economical, and psychological awareness. This process assists individuals to perform self-assessments of power that currently exist within themselves and to maximize control and use of the power they already have to produce a desired outcome.

### 2.2. Historical Context

Gibson [15] emphasizes that an understanding of empowerment is ascertained when there is clarity surrounding its evolution. In the 1960s and 1970s, empowerment in the United States was visualized as community activists supporting the rights of others. The opposite of empower is to exclude or to oppress individuals or groups. African Americans have been known to experience oppression, which is rooted in historical events in America, such as slavery, violations of civil rights, intimidation, and suppression. Suppression was overtly practiced through policies based on white supremacy that produced race-based inequalities that existed politically, socially, and economically [21]. 

Activists such as Martin Luther King fought for equality and justice for all. Although the civil rights movement, led by Dr. King, opened many doors of opportunity for AAs, oppression in the form of poverty, lowered socioeconomic status, and politics remain. However, the battleground on which the fight for equality was fought left scars (real and figurative) in the minds of minorities [21]. These “scars of the mind” within oppressed populations set patterns of thinking that may restrain individuals within such groups from reaching their full potential and attempting to attain equality. For instance, if the mindset exists within individuals or groups that they cannot reach the same status as someone else, then they often will not attempt to try. However, if minds are changed to believe large-scale accomplishments can be attained, then individuals may attempt the challenge. Thus, an empowerment intervention program may facilitate belief in one's abilities.

## 3. Empowerment Research

Empowerment research has been conducted in the disciplines of business, nursing, and psychology. In the literature review which follows, these empowerment studies have been synthesized and grouped into these categories: empowerment in the workplace, empowerment among caregivers, empowerment within communities, and empowerment among AA women. 

### 3.1. Empowerment in the Workplace

Empowerment in the workplace has been examined by researchers to assess its impact on job performance and job satisfaction [22, 23]. A variety of methods was used to empower individuals in these studies. Laschinger [22] focused on respect toward nursing staff from managers and the organization for which they worked. Respect was demonstrated through allowing flexibility in decision-making, fair treatment, and sharing of organizational business matters. In this context, nurses who participated in this shared governance provided better quality of care than those who did not [22]. Furthermore, these nurses, even in stressful situations such as increased patient loads, demonstrated less stress. 

Similarly, Siebert et al. [23] shared integral company information such as company financial status and productivity with organizational employees. Siebert et al. [23] revealed that empowerment through information sharing resulted in greater flexibility and responsibility among workers. Moreover, employees demonstrated enhanced productivity and were happier with their jobs.

### 3.2. Empowerment among Caregivers

Studies to foster empowerment among family (spouses, parents, and children) caregivers have attempted to increase self-care and self-efficacy in their caregiving role, to enhance caregiver coping skills, and to assess knowledge, sense of control, decision-making, and provision of care [11, 24, 25]. In these studies, education was used to achieve targeted caregiver outcomes. Participants received education for varied time periods. Caregivers reported enhanced self-care and self-efficacy [11], as well as demonstrated decreased feelings of dishonor about mental illness, and improvement in family empowerment and coping skills [25]. Another study reported a higher sense of control in caregivers, indicating greater abilities in expressing their needs, accessing information, and making decisions [24].

### 3.3. Empowerment within Communities

Community empowerment studies have attempted to build communal collaboration and participation among community members in order to yield environmental change and improve health [9, 12, 26]. Methods to induce empowerment included small group discussions and training classes, which consisted of varied time frames (ranging from two hours to four years). Two studies found that participants increased communication of what they needed to assist them with environmental changes and improved health [12, 26] while another found that collaboration between community practitioners and constituents within the subjects' neighborhood was demonstrated [9]. These findings suggest that if collaboration between community practitioners and constituents is developed, one may expect that individuals or groups would demonstrate an increase in communication of needs.

### 3.4. Empowerment among African American Women

Education and community participation have attempted to enhance perception of control, self-efficacy, advocacy, and problem-solving abilities in AA women [8, 13, 14, 27]. Community participation consisted of activities such as neighborhood clean-up, crime watch, and the school parent-teacher organization [8] while education included classes with topics, such as introducing concepts of empowerment and self-esteem, talking with grandchildren, and building advocacy skills designed by study participants and researchers, and monthly information sessions “Open Forums” with service providers and community advocates [13, 14]. Findings of these studies demonstrate increased empowerment through the various measures that were used. For instance, Becker et al. [8] found that older participants in the study achieved higher perceived control among those who participated in community activities. Likewise, participants in Joslin's study reported increased life control, self-efficacy, and advocacy. Similarly, Cox [13, 27] found enhanced self-advocacy and coping skills, and as a result, grandmothers became active as community advocates.

### 3.5. Causative Factors

Two causative factors associated with powerlessness include decreased economic and political status. Low socioeconomic status is closely connected with powerlessness [28–30]. Persons with low political status are subjected to discrimination, intolerance, subordination, and stigma, and are marginalized, disenfranchised, and denied human rights [29]. Moreover, people with power are likely in control of political processes, decision-making, and resource distribution while powerless individuals are excluded from such processes [29]. Powerlessness is so insidious that most people subjected to powerlessness do not realize their position, but rather view their lives as the way life is supposed to be. In addition, these victims believe that life cannot be any better because they are consumed with feeling out of control. Freire [31] has described this as “learned helplessness.” He further asserts that those who identify themselves as powerless tend to develop a dependency on those who keep them oppressed. 

Grandparent caregivers with low socioeconomic status and low political status may feel their situations are out of control because they are often forced to assume care for their grandchildren as a result of child neglect and abuse, an incarcerated parent, and parental drug abuse [1]. Therefore, impending stress and health alterations ensue. Moreover, grandparent caregivers are second time around parents and, therefore, experience generational gaps leading to lack of understanding for parenting their grandchildren [32]. These caregivers further report that they need informational resources to assist them in their caregiving roles.

Groups who have limited resources and ensuing risk for disease morbidity and premature mortality are categorized as vulnerable [28, 29]. “Vulnerable groups typically include women and children, ethnic people of color, immigrants, gay men and lesbians, the homeless, and the elderly” [29, page 69]. Thus, AA grandparents providing care for their grandchildren fit the vulnerable definition, and as a result, they often face significant health problems, including depression, coronary artery disease, hypertension, diabetes, and chronic pain in back and joints [5, 33–38]. Clearly, because of health problems and social injustices that AA grandparents face in providing care for their grandchildren, they are considered a powerless and vulnerable group.

## 4. Empowerment Theory and Empowerment Education Framework

### 4.1. Empowerment Theory

Empowerment theory has been used as the framework to develop health education programs to promote the health of individuals and groups for over three decades [13, 27, 31, 39–41]. These studies suggest that knowledge has the power to impact the health of individuals and groups, which is the underpinning for this work. 

One population in need of empowerment is AA grandparents who have assumed the care of their grandchildren. Clearly, grandparents who provide care for grandchildren experience physical and mental health consequences [5, 37, 42–44]. What is not clear are the elements necessary to prevent disease and promote health in this population. Some researchers have suggested that powerlessness contributes to altered disease states [19, 31, 41, 45]. Furthermore, Wallerstein [45] asserts that influence and control over one's life and participation with active groups can be a mediator between stress and health. These researchers recommend empowerment education as a means for improving the health of individuals. Research has identified AA grandmother caregivers as particularly vulnerable to health problems [1, 2, 37, 42–44, 46–48]. Moreover, grandparent caregiving families are at risk of being marginalized [6, 28]. Compounded by an abrupt assumption of caregiving roles, stretched finances, and dysfunctional families, a sense of loss of control over life may ensue. In this context, the effects of an empowerment education program, therefore, may be useful to enhance a sense of control, thereby, minimizing health alterations and stress among this population.

### 4.2. Empowerment Education Framework and Program

**Figure 1:**
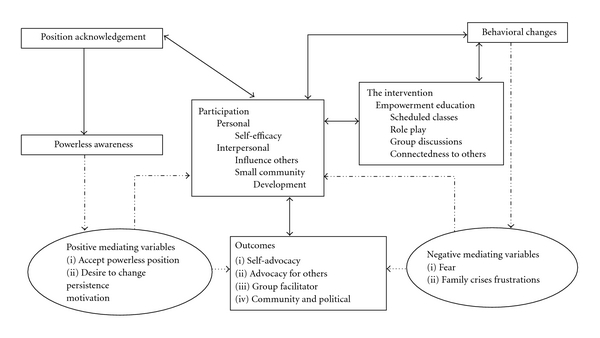
Empowerment education framework.

In this framework, empowerment education is the intervention. A five-concept causal map depicts this hypothesized theory for empowerment education (Figure 1). Primary concepts within this map include powerless position acknowledgement, awareness, participation, behavioral changes, and outcomes. These concepts represent the five phases of the process to become empowered. Mediating variables manipulate desired outcomes once interventions are applied, thus influencing the outcome of a phenomenon [49, 50]. Therefore, even an effective intervention may be influenced by mediating variables.

Mediating variables influence the effects of the intervention. These variables may be positive or negative. Learning how to become empowered often includes attention to (or a focus on) negative mediating variables such as abilities to handle frustrations and disappointments. Frustrations and disappointments may be deterrents and, thus, produce untoward effects. Occasional crises with finances, children, family members, or grandchildren may occur during or after the empowerment process, distracting grandparents, thus altering the learning experience. In addition, once this process is completed, the fear of consequences (embarrassment from responses from individuals in the community or political leaders) from this new behavior may discourage participants from fully developing or performing outcomes such as advocacy.

Conversely, positive mediating variables include desires for change, internal motivators, and persistence, characteristics that are favorable for successful outcome achievement of this empowerment process. While motivation is essential to being persistent for the duration of this empowerment process, participant desire for change is an internal driving force that maintains motivation. Thus, full participation in this empowerment process can be expected from participants who are motivated. Optimal opportunity for outcomes achievement is influenced by the previously mentioned elements.

Acknowledgement of the powerless position is the first phase in gaining empowerment. Individuals must be aware that they are powerless as well as accept this position of powerlessness. Acceptance of powerlessness and the expression of the desire to be different must be present before empowerment can be realized. Once desire to be different is expressed, and participants have demonstrated a readiness to proceed, empowerment education may begin. Realization of the powerless position develops awareness within individuals, which comprises the second phase in the empowerment learning process. Arai [51] suggested that empowerment awareness influences the realization that resources and social connectedness enhance life's satisfaction, which may be a motivating factor (for some) to endure the empowerment process. Awareness also includes identifying with others who are in similar circumstances, seeking resources helpful in developing and enhancing empowerment, and increasing one's own strengths and skills to preserve empowerment. Development of these processes is gained through learning. Moreover, during this learning, connectedness to others, easily available resources, and appropriate support systems enhance the development and maintenance of empowerment.

The third phase in this empowerment process is participation. Participation has been strategically placed in the middle of the model because it influences all concepts. In addition, participation catalyzes, while at the same time, solidifies empowerment. This phase purposes to enhance self-determination through participation [51]. Acquisition of active listening and critical thinking skills is necessary for positioning participants to proceed through this empowerment process successfully [31]. Participation consists of two levels: personal—individual empowerment and self-efficacy—and interpersonal—ability of individuals to influence others [52]. Major goals during this phase are for individuals to develop skills of self-advocacy and advocacy for others. To reinforce attained advocacy skills, grandparent caregivers may participate in small group activities sharing in group discussions and advocating for and collaborating with grandparents with similar experiences in the community. These group activities may be carried out in churches or community service organizations. Further demonstrations of competency may be observed as individuals assist and speak out for others. 

Thus, participation provides opportunities for demonstrating newly acquired empowerment skills. Skills become evident as individuals role play and lead group discussions. Individuals are, therefore, connected, collaborating, and joining alliances [53]. Role playing and group leadership are used to reinforce empowerment skills and to make suggestions when skills are lacking. In addition, collaboration between professionals and individuals is expected to change over the course of the empowerment process. Professionals will exhibit less input (control) and individuals exhibit more input (control) during the participation phase, thus building confidence in decision-making abilities for individuals [19, 53, 54]. Eventually, professionals act as consultants providing supportive guidance. Because participation is an ongoing phase, individuals may fluctuate back and forth between awareness, participation, and change as they learn new skills.

Phase four is characterized by behavioral change in participants' skills and knowledge, which are indicators of their empowerment. These individuals may now be change agents advocating for themselves, their families, and their communities. They may also serve as resources within their communities. 

Finally, in phase five of this empowerment process are the expected outcomes. Outcomes demonstrated are skills in self-advocacy, advocacy for others, group facilitation, and community activism [18, 19, 41]. The assertion is that AA grandparents with these abilities will ultimately demonstrate improved health (decreased morbidities and premature death) through their empowerment [19, 31, 41].

## 5. Implications and Gaps in the Research and Future Recommendations

### 5.1. Implications

Empowered individuals, groups, and communities may demonstrate improved control of their lives, self-efficacy, decision-making, and communication. Thus, individual and community efforts to increase empowerment may bridge gaps between individuals, communities, and practitioners, which may then empower participants to partake more readily in their own well-being, thus decreasing the incidences of disease morbidities and premature death.

Providing care for grandchildren has demonstrated ill-effects on grandparent health. Roe et al. [55] found that AA grandmothers demonstrate worse health outcomes than other ethnic groups. Primarily, health consequences from grandparenting consist of pain, heart problems, and depressed moods [1, 5, 35]. These consequences impact physical, mental, social, and spiritual health of grandparents. Although health alterations have been demonstrated, research is insufficient regarding the impact of empowerment education upon grandparent health. Thus, investigating specific influences that empowerment education may have upon grandparent health is needed. 

Social connectedness or integration is limited in vulnerable populations, thus placing them at higher risk for disease because of lacking communal connections [29]. Communal connections enhance service and information access. Lack of service and information access places individuals at risk for knowledge deficits and subsequently risky behaviors. How will empowerment education help grandmother caregivers? The premise is that advocacy skills developed in this empowerment process will provide opportunities for development of socialization within the community for these grandmothers. Therefore, they may build rapport with individuals as well as businesses, service agencies, and politicians, with whom they can identify. This will, furthermore, build more equitable communal relationships among diverse groups.

### 5.2. Gaps in the Research and Future Recommendations

A significant gap includes insufficient studies that have investigated empowering AA grandmothers; this search effort found only three intervention programs [13, 14, 27]. The shortfall is that Cox [13] and Cox [27] were pilot studies with only 14 AA grandmothers and 11 Latino American grandmother participants. In addition, Cox [13] used the same data from the 14 AA grandmother participants in Cox [27]. Therefore, larger groups of participants are needed. A second gap is the broad focus. Cox [13] designed a curriculum to address the needs of both the grandmothers and the grandchildren. Therefore, to identify the effects of an empowerment education program on grandmother needs more precisely, the education program should only focus on the grandmother. Thus, studies are needed to implement programs to empower AA grandmothers, to investigate their efficacy, and to evaluate their outcomes.

In conclusion, power has not been equally fostered among all people, and the situation of grandparents providing care for grandchildren crosses all ethnic and racial borders. Many problems plague the grandparent caregiver population. Women grandparents, AA grandmothers in particular—since a large number are primary providers for grandchildren—are at an even greater risk for problems subsequent to caregiving, thereby stimulating feelings of powerlessness. Thus, empowerment education programs with health, stress and coping, and self advocacy components may be helpful for all grandparents, regardless of race and ethnicity.

Clearly, AA grandmother caregivers must access power for themselves. To access this power, persons must improve themselves. Self-improvement is developed over time through learning to make better decisions. Decision-making regarding health and other issues that arise may determine well-being. Thus, self-advocacy, advocacy for others, and political activism could promote well thought out decisions for improved health. “Empowerment means creating a space in which a woman can say, it's all right for me to think about myself, not just my family; it's all right to say I need help” [56, page 11]. This empowerment education framework does not claim to be the panacea for those who are powerless, but rather, it hopes to build confidence in AA grandmother caregivers to ultimately positively influence their health. Thus, professionals have an inherent responsibility and a moral calling to empower powerless populations so that these individuals may be able to support self-care abilities while advocating for themselves and others.
